# Editorial: Minimally invasive surgery in gynecology oncology: current trends and controversies

**DOI:** 10.3389/fmed.2023.1353534

**Published:** 2024-01-04

**Authors:** Andrea Giannini, Antonio Simone Laganà

**Affiliations:** ^1^Department of Maternal and Child Health and Urological Sciences, Sapienza University of Rome, Rome, Italy; ^2^Gynecology Division, Department of Medical and Surgical Sciences and Translational Medicine, Sant'Andrea University Hospital, Sapienza University of Rome, Rome, Italy; ^3^Unit of Obstetrics and Gynecology, Department of Health Promotion, Mother and Child Care, Internal Medicine and Medical Specialties (PROMISE), “Paolo Giaccone” Hospital, University of Palermo, Palermo, Italy

**Keywords:** minimally invasive surgery, gynecology oncology, laparoscopy, gynecologic surgery, surgical prospective

The use of Minimally Invasive Surgery (MIS) in Gynecology Oncology is a hot and current topic since its increased use in clinical practice (1); after decades in which laparotomic surgery played the main role in the treatment of gynecologic oncological pathologies, the introduction of “classic” laparoscopy, robotically assisted laparoscopy and vaginal natural transluminal endoscopic orifice surgery (vNOTES) has led to a route inversion in terms of surgical approach (2, 3).

The increased use of MIS is mainly due to the benefits provided by these techniques: less intraoperative blood loss, reduced hospitalization time, faster post-operative recovery, fewer peri and post-operative adverse events and, due to recent innovations, reduced operating costs (1–3). However, the use of minimally invasive techniques is debated and continuously questioned, especially for cervical and ovarian cancers (4–6). The main aim of this Research Topic was to elucidate and delineate the route to follow on a minimally invasive approach, based on current data and future prospective.

Eight high-quality papers were published in this Research Topic: 5 original research, two reviews and one case report.

In the review of Generali et al., the role of MIS for the treatment of ovarian cancer is analyzed in detail. The work focused 4 points: the minimally invasive treatment of early-stage ovarian cancer; the role of laparoscopy as a support technique for upfront surgery planning; the surgical choice for the treatment of advanced tumors after neoadjuvant chemotherapy (MIS vs. open) and the role of laparoscopy for recurrent ovarian cancer. The lack of randomized controlled trials does not allow to define with certainty the possibility of using laparoscopy safely for the treatment of ovarian cancer both for primary debulking surgery (PDS) and for interval debulking surgery (IDS), however, the National Comprehensive Cancer Network (NCCN) and European Society of Medical Oncology (ESMO)–European Society of Gynaecological Oncology (ESGO) guidelines allow the use of this approach for the treatment of the early-stages and recurrent disease. Moreover, the use of laparoscopy for the definition of cytoreducibility (i.e., the possibility to achieve the absence of macroscopically visible tumor) of advanced ovarian tumors is a subject of debate, although some laparoscopic scores are currently used to select patients who would benefit from an upfront surgery compared IDS (7).

As shown in the case report published by Kang D. et al., the minimally invasive approach may be considered safe and effective even for the treatment of recurrent ovarian disease, especially if this recurrence is a mono or oligometastatic disease. In particular, this case focuses on the removal of metastatic disease localized in the spleen root, diagnosed with the evaluation of the tumor marker carbohydrate antigen 125 (CA125) and easily removed using the detail magnification capability allowed by the laparoscopic approach.

Kang J-H. et al. in their original article investigated the efficacy of a continuous wound infiltration (CWI) system for pain management of patients treated with single-port access (SPA) laparoscopy for the presence of adnexal formations. Post-operative pain control is a central issue in gynecological surgery and the use of local (8) and systemic (9) analgesics is the major therapeutic approach for the purpose. The authors investigated the validity of the combined use of these two approaches (fentanyl administered intravenous patient-controlled analgesia plus continuous wound infiltration) and concluded that this method reduces the need of rescue analgesics for the management of post-operative pain in patients treated with SPA.

On the other side, the application of minimally invasive methods for the treatment of endometrial pathology is well established and validated, and such approaches also have an important role in histological and molecular diagnosis. This last aspect was well analyzed by Pados et al. in a review mainly focused on the use of MIS for nodal assessment in endometrial cancer and by Weng et al. in a study conducted on 18 women with endometrial hyperplasia, from which endometrial lavage specimens and parallel biopsy samples were obtained. Indeed, analysis of data obtained from endometrial specimens showed the presence of genetic mutations in 72.7% and 44.4% of women with atypical and non-atypical endometrial hyperplasia, demonstrating that accurate diagnosis is also possible through the use of MIS.

The usefulness of loop electrosurgical excision procedure (LEEP) conization has been investigated in the original article published by Cui et al. that, analyzing data from the treatment of 379 women with papillary squamous cell carcinoma, has demonstrated that LEEP allows a diagnosis with high accuracy, especially if the pre-surgical imaging does not suggest the presence of malignancy.

Macciò et al., reported their experience regarding the outcomes of survival and quality of life (QoL) of 20 patients subjected to pelvic evisceration for advanced-stage gynecologic cancers (7 with open and 13 with laparoscopic approach). Regardless of the method applied for performing the surgery, the authors showed that the QoL of these women was greatly and positively influenced by the surgery itself and, notably, that this also improved survival, highlighting the importance of the spiritual aspect in the care and management of patients with advanced disease and poor prognosis.

Finally, we thought it would be interesting to report the benefits of the application of laparoscopy in the management of heterotopic pregnancies, as demonstrated by Ge et al. in their original, retrospective article in which the results obtained from the surgical removal of the ectopic pregnancy were presented. Sixty-one of the 65 pregnancies were delivered at term without further complications while only 4 resulted in a postoperative abortion. In addition, the application of MIS confirmed the expected results regarding operator time and blood loss.

In conclusion, the introduction of MIS techniques has brought countless advantages to patients with gynecological pathologies, allowing the feasibility of surgery even in fragile patients (10, 11), but many challenges remain regarding the application of MIS for the treatment of gynecological tumors, particularly ovarian and cervical cancers, leaving the need for randomized trials that establish its role and the need of more insights.

We hope that this Research Topic will spark interest of the reader arousing new ideas for future research.

## Author contributions

AG: Writing—original draft, Writing—review & editing. AL: Writing—original draft, Writing—review & editing.
